# Advanced Development of Diverse Photovoltaic-Driven Water Electrolysis for Hydrogen Production: A Review on Coupling Mechanisms, Technological Evolution and Economic Analysis

**DOI:** 10.3390/nano16100579

**Published:** 2026-05-08

**Authors:** Yifei Yu, Suni Shi, Zhiyi Peng, Longlu Wang, Shiyan Wang, Chengbin Liu

**Affiliations:** 1 School of Economics, Nanjing University of Posts and Telecommunications (NJUPT), Nanjing 210023, China; 2 College of Electronic and Optical Engineering & College of Flexible Electronics (Future Technology), Nanjing University of Posts and Telecommunications (NJUPT), Nanjing 210023, China; 3 College of Environment and Resources, Xiangtan University, Xiangtan 411105, China

**Keywords:** photovoltaic-driven water electrolysis, diverse photovoltaic technologies, system integration and coupling, solar-to-hydrogen (STH) efficiency, techno-economic analysis (TEA)

## Abstract

In the context of global carbon neutrality, photovoltaic (PV)-coupled water electrolysis has emerged as a pivotal technological route for large-scale green hydrogen production. This review systematically explores the integration of diverse PV technologies (e.g., crystalline silicon, perovskite tandems, and concentrated PV) with various electrolysis systems (such as AEL, PEMEL, and AEMEL). We analyze the coupling mechanisms across light–electricity–hydrogen multi-energy fields from three dimensions: PV spectral response matching, electrolyzer kinetic adaptation, and innovative system topologies. Furthermore, this paper highlights critical scientific challenges, including the mismatch between fluctuating PV output and steady-state electrolysis, lifecycle stability under extreme conditions, and the optimization of high-cost catalysts. By incorporating cutting-edge approaches like AI-driven predictions, digital twins, and photothermal synergies, we outline future trajectories for enhancing system efficiency and economic viability. Ultimately, this review provides theoretical guidance to advance the commercialization of diverse, stable, and low-cost PV-driven green hydrogen production systems.

## 1. Introduction

Amid global efforts towards carbon neutrality and the restructuring of new energy systems [1,2], hydrogen energy—with its zero-carbon emissions, high energy density, and long-term energy storage capabilities—has emerged as a key enabler for overcoming the bottlenecks in renewable energy integration and achieving deep decarbonization of the energy structure. Photovoltaic-driven water electrolysis (PV-electrolysis) [3,4,5,6], as the mainstream technological pathway for green hydrogen production, directly converts solar energy into hydrogen, thereby achieving a highly efficient “sun-electricity-hydrogen” energy loop. This approach effectively circumvents the carbon emissions associated with traditional fossil fuel-based hydrogen production and the intermittent nature of grid power supply. It has now moved beyond the laboratory R&D stage and entered a critical window period for engineering demonstrations and large-scale application.

Currently, the iterative advancement of photovoltaic technology [7] and the diversified innovation in water electrolysis systems are providing the core driving force for performance breakthroughs and application expansion in photovoltaic hydrogen production systems. On the PV side, mass-produced high-efficiency cell technologies such as N-type TOPCon [8], HJT, and perovskite/silicon tandem [9,10] continue to achieve efficiencies exceeding 26%. New PV architectures, including full-spectrum utilization [11,12], flexible PV [13,14], and concentrated photovoltaics (CPV) [15,16], are constantly emerging, enabling coverage across multiple scenarios—from ground-mounted centralized systems to distributed systems, and from standard environments to extreme operating conditions. On the water electrolysis side, four major technological pathways—alkaline electrolysis (AEL) [17,18], proton exchange membrane electrolysis (PEMEL) [19,20], anion exchange membrane electrolysis (AEMEL) [21,22], and solid oxide electrolysis (SOEC) [23,24]—are developing in parallel. Innovations such as non-precious metal catalysts [25,26], membrane electrode [27,28] assemblies (MEAs), and decoupled water electrolysis (DWE) [29,30] continue to yield breakthroughs [31], effectively balancing the trade-offs between system efficiency, cost, and stability [32].

However, bottlenecks in the multidimensional compatibility between photovoltaic (PV) systems and water electrolysis systems continue to hinder the large-scale development of the industry: First, the intermittent and fluctuating nature of PV output does not align with the steady-state operational requirements of electrolysers. The traditional multi-stage coupling model of “PV–inverter–rectifier–electrolysis” results in significant energy losses, and the matching mechanisms for new topological structures—such as direct DC drive and hybrid coupling—require urgent optimization. Second, a single PV technology struggles to adapt to varying solar resources and application scenarios across different regions; the principles governing the synergistic adaptation of diverse PV technologies (such as crystalline silicon [33,34], perovskite, flexible, and concentrated photovoltaic systems) with different water electrolysis systems remain unclear. Third, system stability and resistance to degradation under extreme operating conditions (such as seawater, high-salinity wastewater, and low-light environments) are insufficient, and issues related to efficiency degradation over the full life cycle and operational and maintenance costs are prominent. Fourth, energy management, intelligent control, and multi-energy complementary technologies at the system integration level lag behind, making it difficult to achieve integrated and efficient synergy among solar, hydrogen, storage, and consumption.

In recent years, academia and industry have conducted in-depth research into the coupled innovation of “diverse photovoltaics and multi-source electrolysis” [35,36,37]. On the one hand, efforts have focused on precisely matching the voltage and power of photovoltaic systems and electrolyzers [38,39]. Through strategies such as direct drive via DC busbars, optimized modular series–parallel configurations, and coordinated energy storage buffering, the conversion efficiency of solar-to-hydrogen (STH) systems has been increased to over 12% [40]. On the other hand, efforts have expanded the application boundaries of PV-based hydrogen production by developing novel systems such as solar–thermal–PV synergy, direct seawater electrolysis, and biomass-assisted co-electrolysis, thereby achieving comprehensive utilization of energy resources and the co-production of high-value-added products; simultaneously, the application of technologies such as artificial intelligence (AI) and digital twins in system modeling, operating condition prediction, and intelligent control has offering insights and potential strategies for addressing the challenge of adapting to PV power fluctuations.

This review adopts an interdisciplinary perspective that integrates diverse photovoltaic technologies with multi-modal water electrolysis systems. It systematically examines the coupling mechanisms, topological architectures, and technical classifications of photovoltaic-driven water electrolysis systems for hydrogen production, with a focus on analyzing the compatibility, performance advantages, and application scenarios of different photovoltaic types (crystalline silicon, perovskite, flexible, concentrated solar power, etc.) with various water electrolysis technology routes. It delves into the core bottlenecks of current systems regarding energy conversion efficiency, operational adaptability, long-term stability, and economic viability. Furthermore, by examining dimensions such as materials innovation, structural optimization, system integration, and intelligent control, the review outlines future development directions for diverse PV-driven water electrolysis hydrogen production systems. The aim is to provide theoretical support and technical references to advance the industrialization of green hydrogen technology and facilitate the energy transition. It should be noted that this review focuses primarily on photovoltaic-driven electrolysis systems, i.e., a technical approach that couples relatively independent photovoltaic modules with electrolyzers via an external circuit. This differs from photoelectrochemical water splitting technology, which typically integrates light absorption and electrochemical reactions into a single device. Although PEC technology holds unique appeal in terms of achieving higher theoretical integration [41,42], the PV-driven electrolysis approach demonstrates significant advantages in terms of scalability across power levels, compatibility with existing energy infrastructure, and modular deployment. This is because it fully leverages the highly mature and independently optimizable PV and electrolysis technologies, making it one of the mainstream solutions for advancing toward large-scale green hydrogen production.

**Figure 1:**
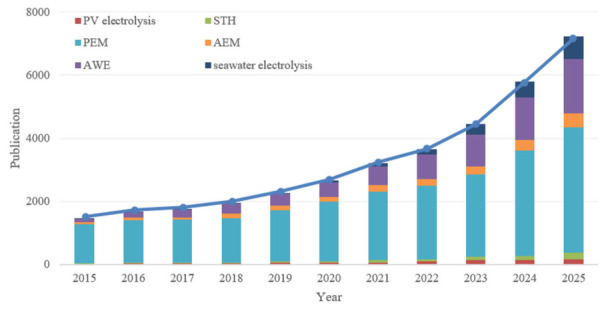
Annual Publication Trends in the Field of Photovoltaic-Driven Electrolytic Hydrogen Production, 2015–2025.

To systematically present the development trajectory and research hotspots in this field, this review conducted a systematic literature search using the Web of Science (WoS) Core Collection database. The search period was set from 2015 to 2025 to capture key advancements over the past decade. The search strategy employed a combination of keywords, including general terms (such as “photovoltaic electrolysis” and “solar-to-hydrogen (STH)”) and specific technical terms (such as “proton exchange membrane (PEM)”, “anion exchange membrane (AEM)”, “seawater electrolysis”, and “alkaline water electrolysis (AWE)”). Figure 1 visually illustrates the annual publication trends revealed by this search: the total number of publications in this field (blue line) shows sustained rapid growth, indicating a significant rise in research activity; the stacked bar chart further analyzes the evolution of interest in different technological pathways. The literature screening focused on peer-reviewed research papers reporting quantitative performance metrics (such as STH efficiency and stability) or demonstrating innovative system integration schemes, to ensure that this review concentrates on work with clear implications for technological advancement.

## 2. System Core Definition and Technical Architecture

A multi-source photovoltaic-driven water electrolysis hydrogen production system is an integrated system that utilizes diverse photovoltaic technologies as its primary power source, integrates various types of water electrolysis units, and achieves efficient conversion of solar energy into hydrogen through power electronics conversion, energy management, and multi-energy complementary control. The core breakthrough lies in addressing the challenges of traditional photovoltaic hydrogen production—namely, low efficiency, high variability, and poor adaptability—by establishing a flexible coupling system comprising multiple photovoltaic sources, multiple electrolyzers, and multiple energy storage units [43].

In recent years, the design of catalytic materials has become increasingly sophisticated. Bimetallic catalysts optimize electronic structures through synergistic effects between their components, while single-atom catalysts enable extremely high atomic utilization and unique active sites; both demonstrate significant potential for enhancing the activity and stability of the oxygen evolution reaction (OER) and hydrogen evolution reaction (HER) [44,45,46]. The key to translating these high-performance catalysts from the laboratory to practical applications lies in integrating them with the actual operating conditions of photovoltaic electrolysis systems, such as high current density and dynamic loading.

The performance and feasibility of such integrated systems depend on several interrelated technical factors. The primary and most fundamental of these is the electrical interface between the photovoltaic (PV) unit and the electrolyzer unit.

The electrical coupling topology between the PV and the electrolyzer critically determines the overall energy transfer efficiency. Direct coupling is simple and low-cost, but if the PV’s maximum power point (MPP) does not match the electrolyzer’s operating voltage, it can result in significant power losses. Coupling via a DC–DC converter allows for voltage regulation, thereby enabling better impedance matching and higher efficiency, albeit at the cost of increased complexity, cost, and conversion losses (typically 2–10%). The most advanced approach involves using a converter with an integrated MPPT, which dynamically tracks the PV’s MPP under varying irradiance to maximize energy harvesting. This is particularly beneficial for large-scale or CPV systems, but it also introduces the highest level of control complexity. The choice of topology represents a critical trade-off between efficiency, cost, and system robustness.

However, the static matching described above is only an ideal scenario. In actual deployment, the system must cope with dynamic operating conditions resulting from the intermittent nature of solar power, which constitutes a second core challenge.

A key and often underestimated challenge is matching under dynamic operating conditions. Actual PV output is inherently intermittent and fluctuating due to cloud cover and the day–night cycle. This causes the electrolyzer to undergo repeated start–stop cycles, rapid load changes, and transient conditions. Such dynamics can lead to significant deviations in efficiency from steady-state values, induce harmful electrochemical stresses (such as reverse current during sudden shutdowns), and accelerate the degradation of catalysts, membranes, and other components due to cyclic mechanical and chemical fatigue. Specifically, the degradation mechanisms of catalysts include oxidation, dissolution, and agglomeration of active sites, as well as poisoning by impurity ions; meanwhile, membrane materials may undergo chemical degradation of polymer chains, mechanical perforation, and loss of ion exchange capacity. Therefore, designing control strategies and materials capable of withstanding these dynamics is just as important as optimizing steady-state performance.

Dynamic electrochemical processes and variable energy inputs interact within a single physical system, introducing a third dimension: thermal management. As a cross-cutting parameter, temperature influences all subsystems, making thermal design critical to system integration.

Temperature is a critical integrated parameter that simultaneously affects all major subsystems. For photovoltaic cells, efficiency typically decreases as temperature rises. Conversely, for electrolyzers, higher temperatures generally enhance reaction kinetics, reduce overpotential, and increase ionic conductivity within the membrane, but may also accelerate material degradation and gas crossover. Furthermore, temperature affects gas solubility and bubble release, thereby influencing mass transfer. This creates a complex optimization problem: waste heat generated by the PV cells or the electrolyzer itself must be managed to balance these contradictory effects—dissipating heat to cool the PV cells and improve their efficiency, while potentially utilizing it to preheat the electrolyte or maintain the stack’s optimal temperature. Consequently, integrated thermal design is crucial for maximizing overall system performance and durability.

Ultimately, losses from electrical coupling, inefficiencies in dynamic operation, and the energy costs of thermal management all accumulate and contribute to the system’s overall energy losses. Consequently, a comprehensive system architecture perspective must extend beyond the core conversion units to examine losses across the entire energy chain.

A realistic assessment of the overall efficiency of a PV–electrolysis system must account for energy losses outside the core PV and electrolysis units. Significant energy is dissipated in the balance-of-system (BOS) components: ohmic losses in interconnections, cables, and connection points; conversion losses in power electronics (DC–DC converters, and inverters if AC-coupled); heat losses from heat sinks and unused waste heat; and energy penalties associated with hydrogen processing, including gas drying, compression, and storage. These ancillary losses can easily consume 10–20% or even more of the initial solar energy. Therefore, the laboratory STH efficiency typically measured at the electrolyzer terminals represents an upper limit. For systems in actual deployment, minimizing BOS losses through efficient component design and system integration is crucial for achieving competitive levelized hydrogen production costs.

Furthermore, the composition of the feedwater imposes fundamental constraints on system design and material selection, particularly when seawater or wastewater is used directly. Chloride ions (Cl^−^) pose a particularly serious threat, as they induce severe corrosion of the anode material and compete with the oxygen evolution reaction (OER) at lower potentials through the chloride evolution reaction (CER), thereby reducing Faradaic efficiency and generating corrosive byproducts. Divalent cations such as Mg^2+^ and Ca^2+^ pose another threat: under localized alkaline conditions, they may form hydroxide or carbonate precipitates on the cathode surface or within the membrane, leading to passivation, increased overpotential, and physical blockage. Therefore, a system designed to treat impure water must either incorporate robust pretreatment steps or utilize materials specifically engineered to tolerate impurities, which constitutes a key axis of technological differentiation.

## 3. Different Types of PV

**Figure 2:**
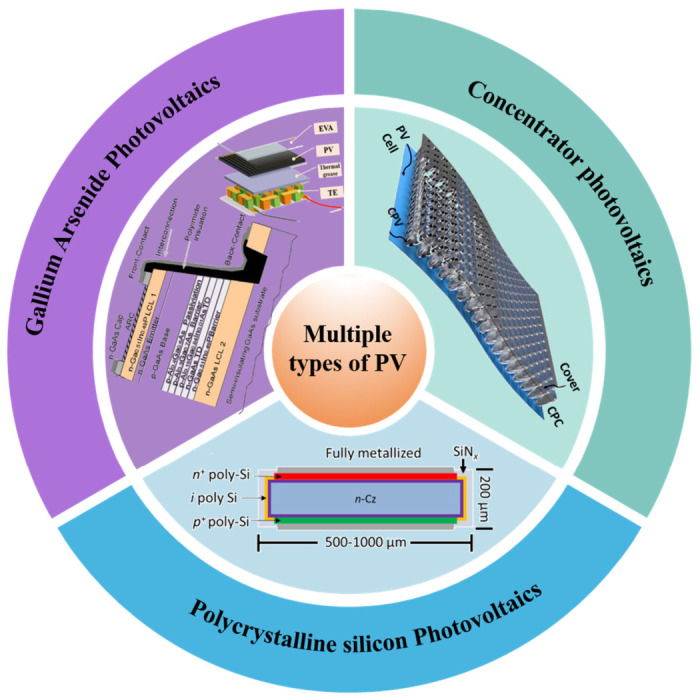
Multiple types of PV.

Crystalline silicon technologies, represented by Passivated Emitter and Rear Cell (PERC), Tunnel Oxide Passivated Contact (TOPCon), and Heterojunction (HJT) [47,48], are the most cost-effective choice for large-scale ground-mounted hydrogen production projects due to their high technical maturity, well-established supply chains, and low costs. With laboratory efficiency exceeding 26% and excellent long-term operational stability, these technologies are ideally suited for integration with alkaline electrolysers (ALK)—which consume significant amounts of electricity and require long-term, reliable operation—to establish stable, large-scale green hydrogen production facilities (Figure 2).

To overcome the theoretical efficiency limit of single-junction photovoltaics, as defined by the Shockley–Queisser limit, tandem photovoltaics [9,49] achieve more complete utilization of the solar spectrum by stacking absorption layers with different bandgaps [50]. For example, the laboratory efficiency of perovskite/silicon tandem cells has exceeded 33%, and III–V multijunction cells (e.g., GaInP/GaAs/Ge) [16] have demonstrated efficiencies over 45% under concentrated sunlight [51]. The key advantage of these technologies lies in their high open-circuit voltage output characteristics, which can better match the high voltage (>1.6 V) required for water splitting. This reduces or even eliminates the energy losses associated with voltage boost conversion, making them an ideal power source for integrated systems targeting the ultimate solar-to-hydrogen (STH) conversion efficiency [52].

CPV technology uses optical lenses or mirrors to concentrate sunlight from a large area onto a small area of high-efficiency multi-junction cells. Its core value lies in replacing the high cost of semiconductor materials with a relatively inexpensive optical system. This makes high-efficiency, high-cost III–V multi-junction cells economically viable. The high current density output of CPV systems perfectly aligns with the need for electrolyzers to increase hydrogen production rates at high current densities. Additionally, the waste heat generated can be used to preheat the electrolyte, achieving “photovoltaic–thermal” synergy and thereby pushing the system’s STH efficiency to new heights [53].

These include flexible thin-film solar cells [54,55,56], building-integrated photovoltaics (BIPV) [57,58,59], and rapidly developing perovskite single-junction cells [60,61]. These technologies are characterized by their lightweight, flexible nature, ability to adapt to low irradiance and non-ideal installation angles, and potential for low-cost manufacturing. They enable distributed, application-specific hydrogen production (such as on rooftops, offshore platforms, and mobile hydrogen production units) and are well-suited for coupling with compact PEM electrolysers to build miniaturized, plug-and-play off-grid hydrogen production systems.

Therefore, the choice of photovoltaic technology is not only a matter of cost and efficiency but also involves spectral and electrical compatibility with the electrolyzer. Water splitting requires a minimum voltage (greater than 1.6 V for liquid water), and higher voltages facilitate the driving of practical current densities with lower resistance losses. Stacked and multi-junction cells (such as perovskite/silicon and III–V compound cells) are particularly attractive because they are designed to capture a broader range of the solar spectrum, convert more photons into usable energy, and inherently provide higher output voltages. This reduces or eliminates the need for voltage-boosting electronic components, directly addressing the challenges of spectral mismatch and insufficient voltage. In contrast, while single-junction silicon cells are cost-effective, their lower voltage output typically requires series connection or DC–DC conversion to reach the required electrolysis potential, thereby introducing additional complexity and potential loss pathways. CPV goes a step further by concentrating light intensity onto ultra-high-efficiency multi-junction cells, effectively delivering both high current and high voltage simultaneously. Therefore, the spectral response and electrical output characteristics of the photovoltaic source are fundamental parameters that must be synergistically optimized with the electrochemical characteristics of the electrolytic cell to achieve a highly efficient system.

## 4. Various Systems

The development of photovoltaic electrolysis systems follows a clear trajectory: from addressing fundamental material challenges to solving complex system-level integration problems. The organization of this section follows this evolutionary process: it begins with systems that emphasize the development of durable catalysts (4.1), transitions to systems incorporating advanced power electronics to achieve electrical matching (4.2), and concludes by presenting cutting-edge systems that achieve deep integration while addressing challenges such as water purification, thermal management, and multi-energy utilization (4.3–4.5). This structure highlights the field’s shift from optimizing individual components to designing holistic, application-oriented solutions.

### 4.1. Photovoltaic–Electrolysis System with Durably Active Catalyst

**Figure 3:**
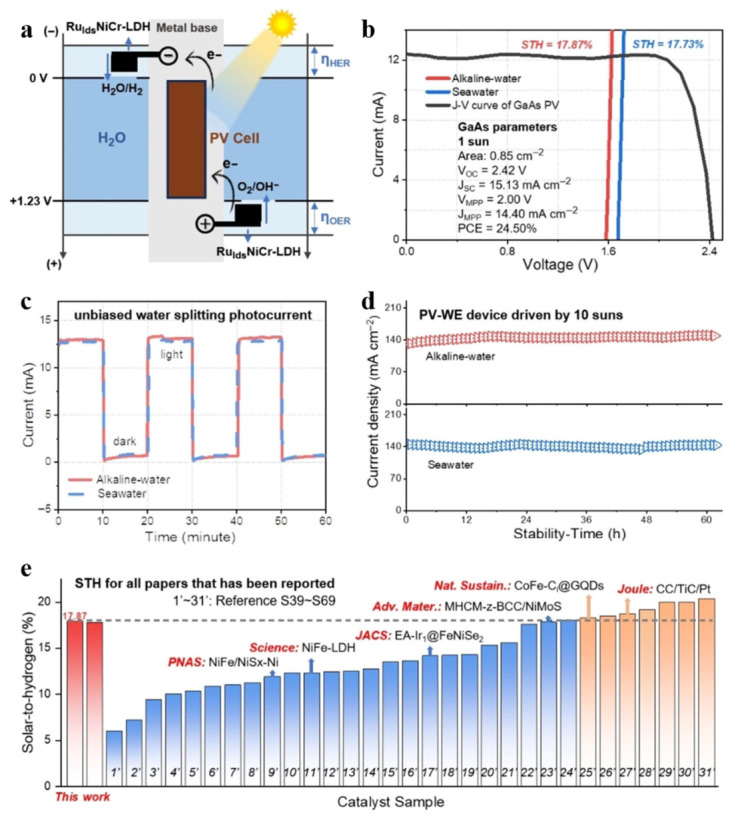
A photovoltaic–electrolysis system consisting of a GaAs solar cell coupled with an electrolyzer based on a Durably Active Catalyst. (**a**) Schematic diagram of the system. (**b**) Solar-to-hydrogen (STH) conversion efficiency of the system in alkaline water and seawater media. (**c**) Instantaneous current response of the system under AM 1.5G standard illumination in the two electrolytes. (**d**) Long-term operational stability test of the system under 10-fold solar irradiance. (**e**) Comparison of the STH efficiency of this work with that of previously reported photovoltaic electrolysis systems. Reprinted with permission from Ref. [62]. Copyright 2024, Wiley.

Wu et al. developed a photovoltaic electrolysis system consisting of a gallium arsenide (GaAs) solar cell coupled with an electrolyzer based on a bifunctional catalyst (Figure 3a) [62]. As shown in Figure 3b, the system achieved solar-to-hydrogen (STH) conversion efficiencies of 17.87% and 17.73% in alkaline water and seawater, respectively, representing a leading performance in this field. Experiments demonstrate that the system can immediately generate a photocurrent under standard illumination in both alkaline water and seawater electrolytes, exhibiting the ability to perform solar-driven water splitting without an external bias voltage (as shown in Figure 3c). Under 10 times the irradiance of sunlight, the system can operate stably for over 60 h, demonstrating excellent long-term operational stability (Figure 3d). Figure 3e clearly demonstrates that, compared to previously reported similar work, the STH efficiency achieved by this system ranks among the top tier. This photovoltaic electrolysis system can efficiently convert renewable energy into hydrogen, and the durable LDH-based catalyst it employs offers a viable prospect for industrial applications.

### 4.2. Photovoltaic–Alkaline Water (PV-AW) Electrolysis System with a Customized DC–DC Converter

**Figure 4:**
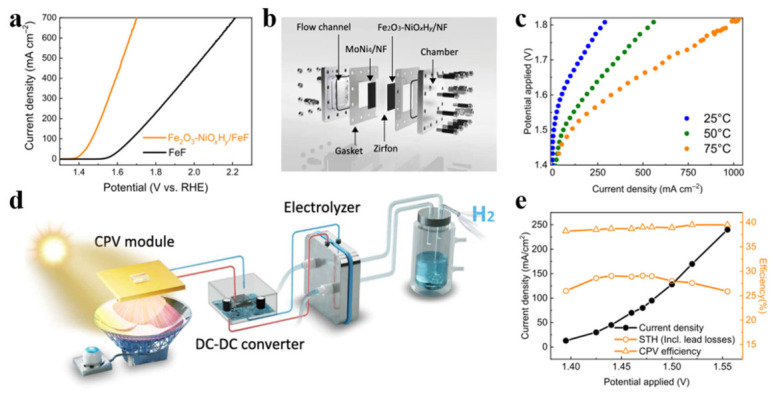
Electrochemical characterization of the Fe_2_O_3_-NiO_x_H_γ_/FeF electrode and its performance in water electrolysis (WE) and photovoltaic–electrolysis (PV-E) systems. (**a**) Polarization curves of different electrodes in 6 M KOH at 25 °C. (**b**) Schematic diagram of a zero-gap alkaline water electrolysis cell. (**c**) Polarization curves of the Fe_2_O_3_-NiO_x_H_γ_/FeF||MoNi_4_/NF electrolysis cell in 6 M KOH at 25 °C, 50 °C, and 75 °C. (**d**) Schematic diagram of the photovoltaic–electrolysis (PV-E) system, which comprises a high-performance Fe_2_O_3_-NiO_x_H_γ_/FeF||MoNi_4_/NF zero-anode-distance alkaline water electrolysis (AWE) cell electro-coupled with a CPV micro-module via a custom DC–DC converter. (**e**) Characteristic I–V curves of the photovoltaic and electrochemical components (measured separately) in the PV-E device, measured at 25 °C, 50 °C, and 75 °C, respectively. Reprinted from Ref. [63].

Photovoltaic–water electrolysis (PV-WE) systems are considered a key solution for large-scale green hydrogen production. However, the solar-to-hydrogen (STH) conversion efficiency of current PV-WE systems generally remains below 20% under practical high current densities, severely limiting their economic viability. Particularly in alkaline water electrolysis (AWE) systems, the sluggish kinetics of the anode oxygen evolution reaction (OER) result in high energy consumption and low electrolysis efficiency. There is an urgent need to develop highly efficient OER catalysts to reduce overpotential and thereby improve the overall energy efficiency of the system. To address this bottleneck, Lu et al. designed a PV-AW system that integrates an alkaline water (AW) electrolyzer with a concentrated photovoltaic (CPV) receiver [63]. They employed a composite catalyst (Fe_2_O_3_-NiO_x_H_y_), consisting of ultrafine iron oxide nanoparticles supported on hydroxylated nickel oxide nanosheets, as the anode oxygen evolution catalyst, which demonstrated significantly superior performance compared to a single Fe_2_O_3_ catalyst. The resulting large-area electrodes were adapted for use in electrolyzer devices, enabling the device to achieve a hydrogen production current density of 1 A cm^−2^ at a cell voltage of 1.8 V. The integrated PV-AWE system is shown in Figure 4. In this system, the zero-pitch AWE tank is connected to a CPV micro-module via a custom DC–DC converter. High-reflectivity concentrating mirrors focus natural sunlight onto a triple-junction solar cell (GaInP/GaInAs/Ge) mounted on a water-cooled heat sink, with sunlight alignment controlled by a sun tracker. This system achieved a solar-to-hydrogen (STH) conversion efficiency of up to 29.1% at high current densities, comprehensively surpassing all previously reported PV-AWE systems. It is worth noting that achieving this record-breaking efficiency requires operation at extremely high current densities (~1 A cm^−2^), which may place additional strain on the durability of the catalyst and membrane. This presents a classic example of an efficiency–stability trade-off; in practical applications, pushing the performance limits must be carefully balanced against long-term operational robustness.

### 4.3. Plug-and-Play Solar-Powered Membrane Distillation–Electrolysis System

**Figure 5:**
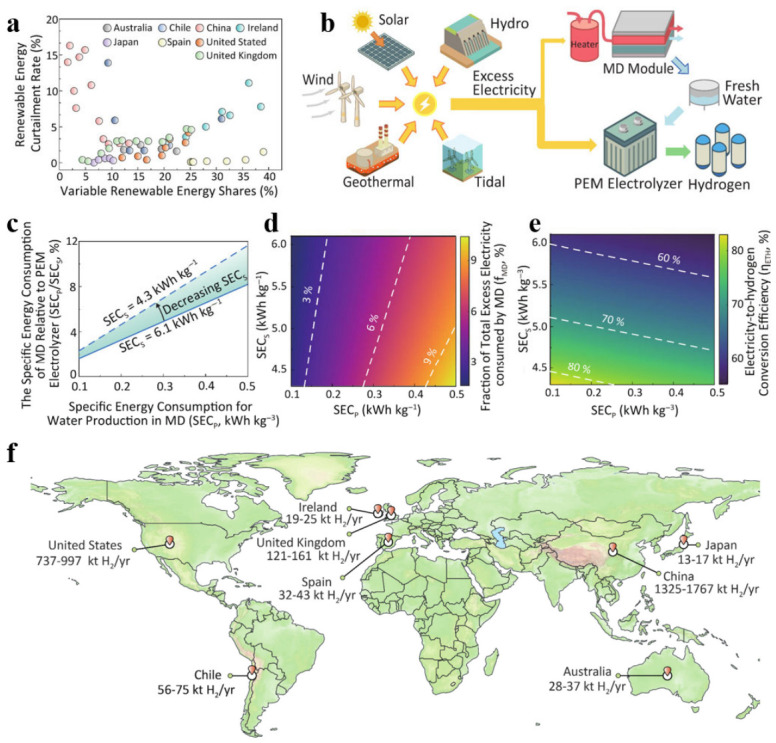
The potential of solar-driven membrane distillation–electrolysis (SMDE) systems for absorbing surplus renewable energy. (**a**) The trend showing that the curtailment rate of renewable energy increases as its penetration into the grid rises. (**b**) A schematic diagram illustrating how the SMDE system functions as a dispatchable load. (**c**) Comparison of specific energy consumption between the membrane distillation (MD) water production process and the proton exchange membrane (PEM) electrolysis hydrogen production process. (**d**) Proportion of surplus electricity allocated to the MD process. (**e**) Overall conversion efficiency (η_ETH) of the SMDE system from surplus electricity to hydrogen. (**f**) Estimated global hydrogen production potential based on global curtailment data and an assumed η_ETH of 60–80%. Reprinted with permission from Ref. [64]. Copyright 2026, American Chemical Society.

Lin et al. proposed a plug-and-play solar-driven membrane distillation–electrolysis (SMDE) system that achieves the synergistic conversion of seawater into high-purity freshwater and hydrogen by directly integrating photovoltaic panels, membrane distillation modules, and proton exchange membrane electrolysers [64]. Figure 5 illustrates the potential and operational logic of the solar-powered membrane distillation–electrolysis (SMDE) system for absorbing surplus renewable energy. As shown in Figure 5a, as the penetration rate of renewable energy increases, the rate of curtailed power on the grid rises accordingly, forming the practical context for this study. To address this issue, the proposed solution operates as illustrated in Figure 5b. Functioning as a dispatchable load, the system allocates surplus electricity to the membrane distillation (MD) unit for freshwater production and to drive the proton exchange membrane (PEM) electrolyzer for hydrogen generation. Figure 5c illustrates a comparison of energy consumption, showing that the low specific energy consumption of MD water production (0.1–0.5 kWh kg^−1^) is significantly lower than that of PEM electrolysis (4.3–6.1 kWh kg^−1^). The system’s power distribution (Figure 5d) shows that less than 11% of the surplus electricity is required to meet the entire freshwater demand, allowing over 90% of the electricity to be directly used for high-efficiency electrolysis. This enables the system’s overall electricity-to-hydrogen conversion efficiency (η_ETH) to remain high, reaching 55% to 83% as shown in Figure 5e. Finally, an assessment of global potential based on this efficiency (Figure 5f) quantifies the massive scale at which this technology pathway can utilize curtailed electricity to produce hydrogen, providing direct evidence that it is a viable solution for renewable energy storage and grid regulation.

### 4.4. Interfacial Solar Vapor Electrolyzer for Efficient and Durable Hydrogen Production Directly from Seawater

**Figure 6:**
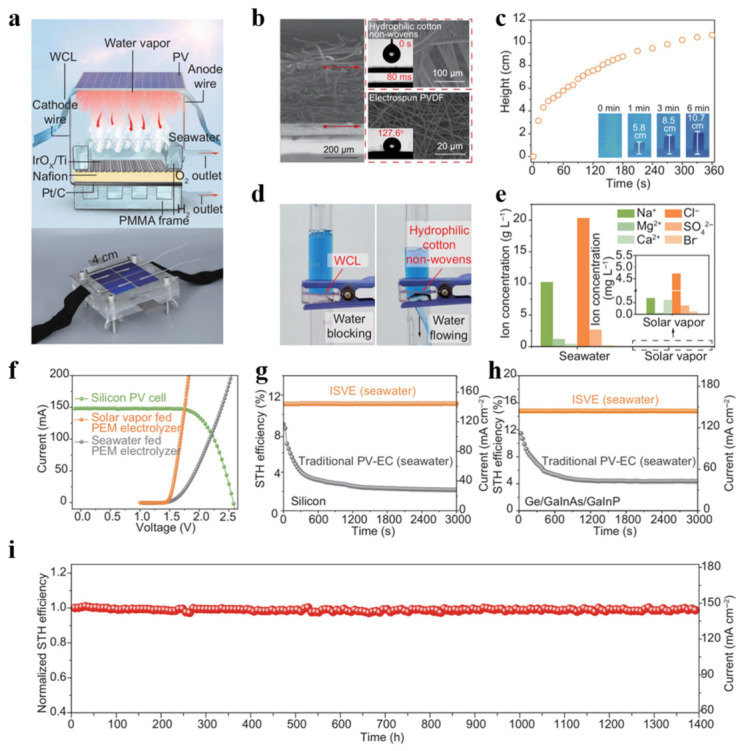
Interface Solar Vapor Electrolyzer (ISVE) for direct hydrogen production from seawater. (**a**) Schematic and photograph of the ISVE device. (**b**) Scanning electron microscope images of the key component—the water supply and anti-contamination layer (WCL). (**c**) Image demonstrating the capillary water absorption performance of the WCL (water droplets are completely absorbed within 80 milliseconds). (**d**) Image showing the hydrophobic surface of the WCL preventing seawater droplet penetration. (**e**) Comparison of concentrations of harmful ions (Na^+^, Mg^2+^, Cl^−^, etc.) in raw seawater versus interface solar vapor generated by the ISVE. (**f**) J-V curve of a silicon-based photovoltaic cell under 1-sun illumination, and initial polarization curves of a PEM electrolyzer fed with solar vapor and seawater, respectively. (**g**) STH efficiency and current density of the silicon PV-based ISVE system and the conventional PV-EC system under simulated 1-sun irradiation with direct seawater inflow. (**h**) STH efficiency and current density of the high-efficiency Ge/GaInAs/GaInP PV-based ISVE system and the conventional PV-EC system under the same conditions. (**i**) Long-term operational durability test results of the ISVE under 1-sun irradiation (performance is expressed as normalized STH efficiency and current density). Reprinted from Ref. [65].

Zhu et al. designed an interfacial solar vapor electrolyzer (ISVE) for the direct production of hydrogen from seawater. Its core design and performance validation are systematically presented in Figure 6 [65]. The overall architecture and operating principle of the device are illustrated in the schematic shown in Figure 6a. The system consists of a photovoltaic cell, a unique water supply and anti-contamination layer (WCL), and a proton exchange membrane (PEM) electrolyzer, all tightly coupled together. The photovoltaic cell not only generates electricity to drive electrolysis, but its waste heat is also utilized by the WCL to produce purified thermal steam, which is then fed into the electrolyzer for decomposition. The microstructure of the WCL is revealed by the scanning electron microscope image in Figure 6b. It consists of a hydrophilic cotton fabric covered with electrospun PVDF nanofibers. This design confers critical wetting and barrier properties to the WCL. Its ultra-fast capillary water absorption (Figure 6c) ensures a continuous water supply, while its hydrophobic surface effectively prevents leakage of liquid seawater (Figure 6d). More importantly, the steam produced through this process is of extremely high purity; as shown in the ion concentration comparison in Figure 6e, the concentration of harmful ions in the steam is reduced by several orders of magnitude compared to that in the original seawater. A significant reduction in the concentration of harmful ions, particularly chloride ions (Cl^−^), is crucial. This alleviates two major challenges associated with direct seawater electrolysis: (1) corrosion of anode materials (such as Ni and Ir) by Cl^−^; (2) the competitive chloride evolution reaction (CER), which may occur at a potential lower than that of the oxygen evolution reaction (OER), thereby reducing Faradaic efficiency and generating corrosive chlorine gas. The near-complete elimination of scaling ions, such as magnesium ions (Mg^2+^) and calcium ions (Ca^2+^), also prevents the formation of insulating deposits on the electrode surfaces. In terms of performance, the electrochemical operating point of the system was determined by comparing the polarization curves in Figure 6f, indicating that the ISVE mode exhibits superior voltage characteristics compared to conventional direct seawater electrolysis. The efficiency advantage is quantified in Figure 6g. The silicon PV-based ISVE achieved a solar-to-hydrogen (STH) conversion efficiency of approximately 11.1%, significantly outperforming the control system. When combined with high-efficiency III–V multi-junction PV cells, the STH efficiency can be further increased to 15.2% (Figure 6h). Finally, long-term operation tests (Figure 6i) confirmed the system’s exceptional stability, with continuous hydrogen production using seawater as the feedstock for over 1400 h without significant degradation, demonstrating the effectiveness of this integrated design in addressing corrosion and pollution issues.

### 4.5. Hybrid Solar Distillation–Water Electrolysis (HSD-WE) Device

**Figure 7:**
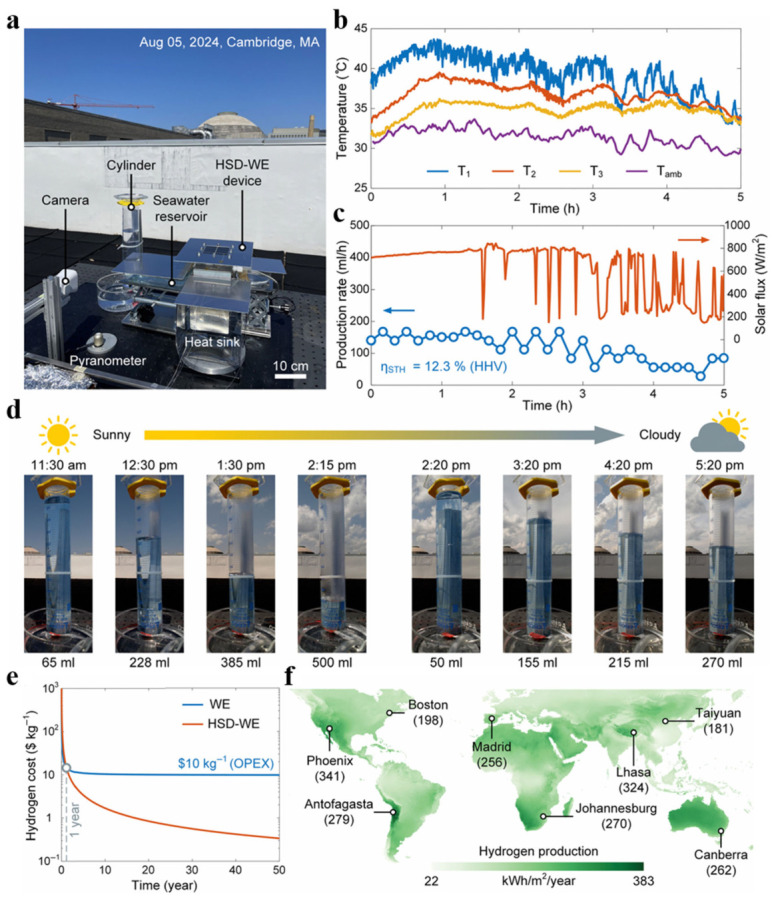
Results of outdoor testing and a techno-economic analysis of the hybrid solar distillation–water electrolysis (HSD-WE) system. (**a**) Photograph of the outdoor experimental setup installed on a rooftop. The test was conducted on August 5, 2024, under partly cloudy conditions in Cambridge, Massachusetts, USA. (**b**) Temperature-versus-time curves for key components of the HSD-WE system during the outdoor test. (**c**) Relationship between solar irradiance (red line) and measured green hydrogen yield (blue line) over time. Hydrogen yield was determined using the drainage method. During the test, the average STH efficiency of the HSD-WE system, calculated based on the higher heating value (HHV), was 12.3%. (**d**) A time-lapse image of the HSD-WE unit continuously producing green hydrogen during the outdoor test. (**e**) The trend of hydrogen production cost over system operating time. The cost of hydrogen production via conventional water electrolysis (WE) is constrained by operating expenses (OPEX) associated with the consumption of clean water and electricity. The cost of hydrogen production using the HSD-WE technology pathway falls below that of conventional WE after one year of operation, reaching $5/kg after three years and $1/kg after fifteen years. (**f**) Map showing the projected distribution of global green hydrogen production potential using the HSD-WE method. Using verified STH efficiency as an input parameter, the projected average annual green hydrogen production is 233 kWh m^−2^. The map indicates green hydrogen production in selected cities worldwide; the values in parentheses represent the annual production per unit area for each city, in kWh m^−2^/year. Reprinted from Ref. [66].

Zhang et al. developed a hybrid solar distillation–water electrolysis (HSD-WE) system that integrates photovoltaic power generation with solar thermal distillation to achieve full-spectrum utilization of solar energy, thereby efficiently producing green hydrogen using only sunlight and seawater as inputs [66]. The core performance validation, economic evaluation, and global application potential of this system are systematically presented in Figure 7. A photograph of the system’s outdoor experimental setup is shown in Figure 7a. During testing, the temperature trends at key locations within the setup are shown in Figure 7b, indicating that the temperature of the HSD-WE system rose rapidly within one hour. In terms of performance, Figure 7c simultaneously displays the measured solar irradiance and the corresponding green hydrogen production rate, from which an average solar-to-hydrogen (STH) conversion efficiency of 12.3% was calculated. The continuity and stability of the gas production process are clearly demonstrated in Figure 7d. The time-series graph, together with the yield data, indicates that the system’s hydrogen production rate remained above 100 mL h^−1^ until 3:00 PM, when solar irradiance dropped significantly. The techno-economic analysis in Figure 7e compares the hydrogen production costs of the HSD-WE pathway with those of conventional electrolysis. The results show that the former achieves a cost advantage after just one year of operation and can be significantly reduced to $1/kg over the long term. Finally, the spatial distribution of the global annual green hydrogen production potential based on system efficiency simulations (Figure 7f) further highlights the prospects for large-scale application. The figure clearly indicates the annual hydrogen production per unit area for several representative cities worldwide.

### 4.6. System Comparison

The representative systems discussed in Section 4.1, Section 4.2, Section 4.3, Section 4.4 and Section 4.5 demonstrate various strategies for advancing photovoltaic electrolysis technology. To synthesize their characteristics and provide a clear cross-comparison, Table 1 summarizes their key performance metrics, materials, and integration methods. The comparison yields the following insights: (i) Systems aimed at achieving extreme STH efficiency (such as the CPV-AWE system in Section 4.2) typically employ complex, high-cost components and may face durability challenges under extreme operating conditions. (ii) Systems designed for exceptional durability and operation with impure water (such as the ISVE in Section 4.4) achieve significant stability through the integration of auxiliary processes like in situ purification, but sometimes involve trade-offs in system simplicity and initial efficiency. (iii) There are significant variations in technology readiness, indicating that while laboratory breakthroughs are promising, achieving commercialization requires concerted efforts in scaling up, cost reduction, and long-term field testing. This comparison underscores that there is no universally optimal design; selection depends largely on specific application requirements, such as local solar resources, water availability, and priorities regarding efficiency, cost, or durability.

Based on the comprehensive comparison in Table 1 and the case studies discussed above, we can clearly identify the characteristics, suitable applications, and challenges faced by different technological approaches.

Systems designed to achieve maximum STH efficiency, such as the PV-AW system based on III–V multi-junction CPV (Section 4.2), represent the pinnacle of current technology with an efficiency of 29.1%. The target application for such systems is large-scale centralized hydrogen production plants in regions with abundant solar resources (high direct irradiance). However, the main challenges lie in the high cost of CPV modules, the complexity of the concentrating and sun-tracking systems, and long-term thermal management and durability issues during operation at high current densities.

Systems designed for practical water environments and high stability requirements, such as the ISVE (Section 4.4) and the Rulds NiCr-LDH-based PV-EC system (Section 4.1), demonstrate strong adaptability. By utilizing waste heat to generate pure steam, ISVE has achieved ultra-long-term stable operation exceeding 1400 h. Its key feature is inherent resistance to fouling, and its target applications include distributed hydrogen production scenarios in coastal and island regions where freshwater is scarce but seawater resources are abundant. The challenges lie in the design of the steam–electrolysis coupling interface and the optimization of water transport. In contrast, the PV-EC system directly electrolyzes seawater using an innovative bifunctional catalyst, maintaining stability at an efficiency of ~17.7%; its cost is primarily constrained by the current price of GaAs photovoltaic cells.

Solutions dedicated to system integration innovation and energy synergy, such as SMDE (Section 4.3) and HSD-WE (Section 4.5), offer distinct value propositions. The SMDE system combines membrane distillation with electrolysis; its key feature is the ability to utilize curtailed renewable energy and waste heat from PV systems. Its target application is to serve as a dispatchable load for grid regulation. Its commercialization costs and primary challenges are closely related to the long-term anti-fouling performance of MD membranes and system energy consumption. The HSD-WE system demonstrates the feasibility of full-spectrum utilization in outdoor settings, producing freshwater as a byproduct while generating hydrogen. This reduces reliance on infrastructure and makes it suitable for distributed deployment. Its future development must overcome challenges such as cost optimization through integration and verification of long-term outdoor reliability.

**Figure 8:**
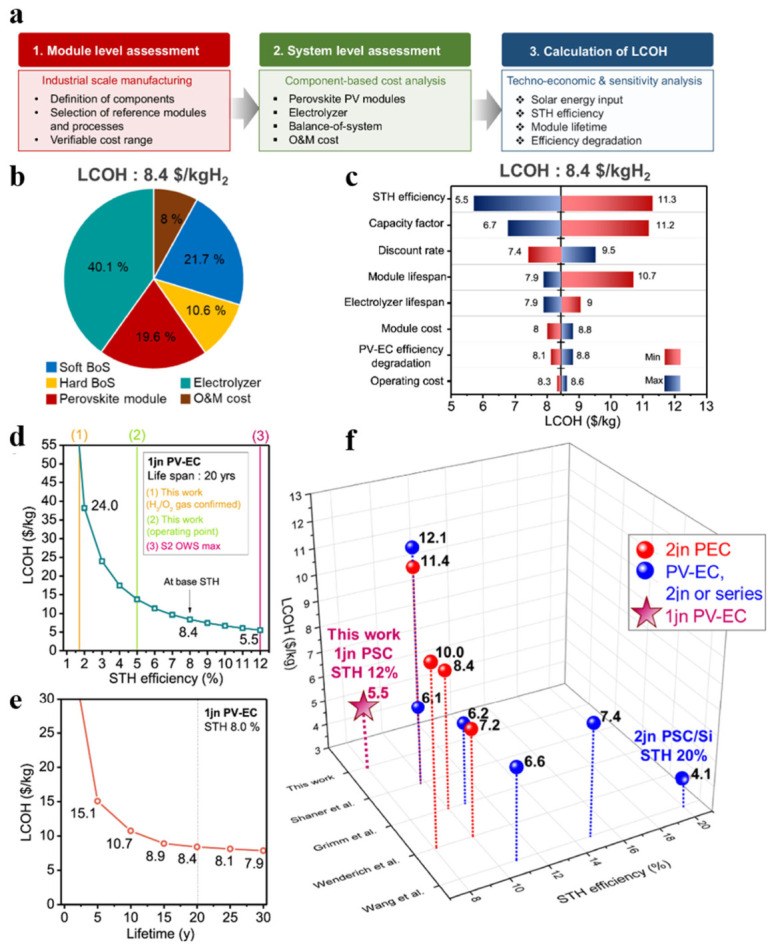
The technical–economic analysis (TEA) framework and key findings for a photovoltaic seawater electrolysis hydrogen production system. (**a**) Standard flowchart of the TEA analysis. (**b**) Cost breakdown diagram for the levelized cost of hydrogen (LCOH). (**c**) Results of a sensitivity analysis using several key technical and economic parameters. (**d**) LCOH trend estimated based on the solar-to-hydrogen (STH) efficiency obtained in this study (1.7% measured by gas evolution method and 5.0% measured by operating point reading method) and the maximum theoretical STH efficiency (12%) of an S2-type overall water splitting (OWS) system with an assumed 20-year lifespan. (**e**) Analysis of the impact of system module lifespan on LCOH, assuming a fixed STH efficiency of 8.0%. (**f**) Results of a comparative economic performance analysis between this technology and other representative works, using LCOH and η_STH as metrics, based on literature data. Reprinted from Ref. [67].

Finally, TEA studies on emerging material systems (such as perovskites) (Figure 8) indicate that while pursuing higher efficiency, material cost and lifespan are the most sensitive variables affecting LCOH, pointing the way toward economic viability for future technological R&D.

## 5. Economic and Technical Analysis

In the process of advancing solar-driven hydrogen production technology from the laboratory to commercial application, technical and economic analysis (TEA) plays a crucial role. It serves not only as a bridge linking technical performance (such as STH efficiency and stability) to ultimate market competitiveness (levelized cost of hydrogen, LCOH), but also as a key tool for identifying technical bottlenecks and guiding R&D directions. However, in most current studies, TEA remains relatively simplistic, often relying on idealized assumptions or overlooking critical cost components and scaling challenges, resulting in conclusions of limited practical value.

In contrast, Kim et al. conducted a relatively comprehensive techno-economic analysis of a photovoltaic water electrolysis system for hydrogen production based on CsPbBr_3_ solar cells. Figure 8 presents the techno-economic analysis (TEA) framework and key conclusions for a highly efficient and stable photovoltaic seawater electrolysis system for hydrogen production [67]. As shown in the flowchart in Figure 8a, this analysis followed a standard procedure ranging from the definition of technical parameters and the construction of cost models to the calculation of the levelized cost of hydrogen (LCOH). The cost breakdown diagram in Figure 8b specifically illustrates the detailed composition of the LCOH, with the electrolyzer system (including catalysts, membranes, etc.) and the perovskite solar cells constituting the primary cost components; the LCOH under baseline conditions is calculated to be $8.4/kg. The results of the sensitivity analysis (Figure 8c) clearly reveal the extent to which various technical and economic parameters influence LCOH, indicating that the solar-to-hydrogen conversion efficiency (η_STH) is the parameter most sensitive to economic performance, followed by the capacity factor (i.e., solar availability), the discount rate, and the module lifetime. This relationship is further quantified in Figure 8d, which shows that LCOH decreases significantly as η_STH increases, reaching a critical level of approximately $5.5/kg when the system achieves its theoretical maximum efficiency of 12%. Figure 8e examines the impact of system module lifespan on LCOH under the assumption that η_STH is fixed at 8.0%. Finally, as shown in the economic performance comparison in Figure 8f, this study compares the estimated LCOH and η_STH with other representative works in the literature, thereby positioning the potential economic competitiveness of this technology within a broader context.

It is crucial to interpret TEA results in light of a clear understanding of their underlying assumptions and uncertainties. As shown in Figure 8c, the predicted LCOH is highly sensitive to several input parameters. The assumed solar-to-hydrogen (STH) conversion efficiency typically has the greatest impact; even minor changes can drastically affect annual hydrogen production. Parameters such as system lifespan, discount rates, and local solar irradiance (capacity factor) also introduce significant uncertainty, and their values can vary widely depending on technological advancements, financial conditions, and geographic location. Furthermore, many TEA studies assume that system performance remains stable over several decades, which may not fully account for unforeseen degradation rates, maintenance costs, or performance losses during scaling up. Therefore, LCOH values should be viewed as indicative ranges under specific boundary conditions, and comparisons between different studies require careful alignment of these underlying assumptions.

## 6. Conclusions and Perspective

In the process of transitioning the global energy structure toward a clean, low-carbon future, diverse photovoltaic-driven water electrolysis systems have emerged as one of the most promising technological pathways for the large-scale production of green hydrogen, thanks to their ability to achieve direct “sunlight-to-electricity-to-hydrogen” conversion. This review systematically examines strategies for the cross-integration of various technologies—ranging from high-efficiency crystalline silicon [33,68], tandem photovoltaics, and concentrated photovoltaics to flexible photovoltaics—with diverse electrolysis systems, including alkaline, proton exchange membrane, and anion exchange membrane systems. Research indicates that the evolution of these systems follows a clear trajectory from “separate coupling” to “monolithic integration,” with the core objective being to synergistically enhance the conversion efficiency from solar energy to hydrogen, long-term operational stability, and economic viability. However, current technologies still face key challenges, including the mismatch between the intermittent nature of PV output and the steady-state operational requirements of electrolyzers, insufficient material and system stability under complex water source conditions, and the difficulty of simultaneously achieving high efficiency and low cost.

A key frontier lies in addressing the challenges of scaling up. The transition from small, uniform laboratory cells to industrial-scale stacks and plants introduces non-ideal conditions such as current and temperature gradients across large-area electrodes, uneven illumination in vast photovoltaic fields, and increased complexity in fluid distribution and gas management. These factors can lead to local hotspots, accelerated degradation, and efficiency losses not observed at smaller scales. Therefore, future research must bridge this gap by conducting studies on multi-cell stacks and integrated pilot-scale plants, and by developing models that account for these scaling effects, to ensure that the promising efficiencies reported in the laboratory can be translated into reliable, high-efficiency large-scale systems.

Looking ahead, overcoming these bottlenecks requires collaborative innovation at the material, device, and system levels. At the materials level, efforts should focus on developing non-precious metal catalysts that combine high activity with excellent durability, and on designing dynamic protective interfaces capable of withstanding counter-current and impurity ion impacts. At the system architecture level, intelligent energy management and coupling topologies must be developed to utilize artificial intelligence for predicting power fluctuations and achieving synergistic optimization of multi-energy flows (light–electricity–heat), thereby maximizing overall system efficiency; the integration of PV-E systems into hybrid renewable energy platforms, combined with wind power or storage, represents a key application in this direction, effectively mitigating power intermittency and enhancing system economics and reliability. More importantly, it is essential to establish a refined techno-economic analysis (TEA) model covering the entire lifecycle, integrating the evaluation of core parameters such as efficiency, lifespan, and costs. To ensure meaningful comparisons, it is equally crucial to promote standardized performance assessment protocols, including consensus on reporting STH efficiency (based on HHV or LHV) and stability testing standards. This will clarify the commercialization boundaries and cost-reduction pathways for different technological routes. Only through such multidimensional, cross-scale integrated innovation can photovoltaic hydrogen production technology transition from demonstration to large-scale, economically viable industrial deployment, ultimately providing a solid foundation for a sustainable hydrogen economy.

## Figures and Tables

**Table 1:** Comparison of Photovoltaic-Driven Water Electrolysis Systems for Hydrogen Production Based on Spectral Coupling Mechanisms.

System	PV Technology	Electrolyzer Type	Water Feed /Electrolyte	STH Efficiency(HHV)	Current Density	Stability (Hours)	Ref
PV-EC with RuldsNiCr-LDH	GaAs single-junction	Custom AEM electrolyzer (Ru_lds_NiCr-LDH bifunctional catalyst)	Alkaline water/ Seawater	17.87% (AW)/ 17.73% (SW)	~15 mA cm^−2^ (1 sun)	>60 (10 Suns)	[62]
PV-AW with CPV	GalnP/GalnAs/Ge (Concentrator PV, CPV)	Zero-gap Alkaline Water Electrolyzer (AWE)	6 M KOH	29.1%	~280 mA cm^−2^ (@1.8V)	-	[63]
SMDE System	Conventional c-Si	Proton Exchange Membrane (PEM) electrolyzer	Seawater (via MD)	System η_ETH: 55–83%	-	-	[64]
ISVE System	Si-based/III–V multi-junction	Proton Exchange Membrane (PEM) electrolyzer	Seawater (Vapor)	11.1% (Si)/ 15.2% (III–V)	~144/~148 mA cm^−2^	>1400	[65]
HSD-WE System	Si-based (Integrated)	Proton Exchange Membrane (PEM) electrolyzer	Seawater	12.3% (outdoor average)	-	One-day test (stable)	[66]
TEA Case Study	CsPbBr_3_ Perovskite	Not specified	Not specified	5.0% (operating point)	-	- (Model assumption)	[67]

Note: The STH efficiencies are reported as per the original references. Most are based on the higher heating value (HHV) of hydrogen. Stability test conditions (current density, temperature, illumination continuity) vary across studies. “-” indicates that the data was not provided in the reference.

## Data Availability

No new data were created or analyzed in this study.
